# Series: Practical guidance to qualitative research. Part 1: Introduction

**DOI:** 10.1080/13814788.2017.1375093

**Published:** 2017-11-29

**Authors:** Albine Moser, Irene Korstjens

**Affiliations:** a Faculty of Health Care, Research Centre Autonomy and Participation of Chronically Ill People, Zuyd University of Applied Sciences, Heerlen, The Netherlands;; b Faculty of Health, Medicine and Life Sciences, Department of Family Medicine, Maastricht University, Maastricht, The Netherlands;; c Faculty of Health Care, Research Centre for Midwifery Science, Zuyd University of Applied Sciences, Maastricht, The Netherlands

**Keywords:** Qualitative research, qualitative methodology, phenomena, natural context, emerging design, primary care

## Abstract

In the course of our supervisory work over the years, we have noticed that qualitative research tends to evoke a lot of questions and worries, so-called Frequently Asked Questions. This journal series of four articles intends to provide novice researchers with practical guidance for conducting high-quality qualitative research in primary care. By ‘novice’ we mean Master’s students and junior researchers, as well as experienced quantitative researchers who are engaging in qualitative research for the first time. This series addresses their questions and provides researchers, readers, reviewers and editors with references to criteria and tools for judging the quality of papers reporting on qualitative research. This first article describes the key features of qualitative research, provides publications for further learning and reading, and gives an outline of the series.

## Introduction

In the course of our supervisory work over the years, we have noticed that while many researchers who conducted qualitative research for the first time understood the tenets of qualitative research, knowing about qualitative methodology and carrying out qualitative research were two different things. We noticed that they somehow mixed quantitative and qualitative methodology and methods. We also observed that they experienced many uncertainties when doing qualitative research. They expressed a great need for practical guidance regarding key methodological issues. For example, questions often heard and addressed were, ‘What kind of literature would I search for when preparing a qualitative study?’ ‘Is it normal that my research question seems to change during the study?’ ‘What types of sampling can I use?’ ‘What methods of data collection are appropriate?’ ‘Can I wait with my analysis until all data have been collected?’ ‘What are the quality criteria for qualitative research?’ ‘How do I report my qualitative study?’ This induced us to write this series providing ‘practical guidance’ to qualitative research.

## Qualitative research

Qualitative research has been defined as the investigation of phenomena, typically in an in-depth and holistic fashion, through the collection of rich narrative materials using a flexible research design [1]. Qualitative research aims to provide in-depth insights and understanding of real-world problems and, in contrast to quantitative research, it does not introduce treatments, manipulate or quantify predefined variables. Qualitative research encompasses many different designs, which however share several key features as presented in Box 1.

Box 1. Key features of qualitative research.

**Table:** 

Qualitative research studies phenomena in the natural contexts of individuals or groups.
Qualitative researchers try to gain a deeper understanding of people’s experiences, perceptions, behaviour and processes and the meanings they attach to them.
During the research process, researchers use ‘emerging design’ to be flexible in adjusting to the context.
Data collection and analysis are iterative processes that happen simultaneously as the research progresses.

Qualitative research is associated with the constructivist or naturalistic paradigm, which began as a countermovement to the positivistic paradigm associated with quantitative research. Where positivism assumes that there is an orderly reality that can be objectively studied, constructivism holds that there are multiple interpretations of reality and that the goal of the research is to understand how individuals construct reality within their natural context [1].

## High-quality qualitative research in primary care

Qualitative research is a vital aspect of research in primary care and qualitative studies with a clear and important clinical message can be highly cited [2,3]. This series intends to provide novice researchers an introduction to information about conducting high-quality qualitative research in the field of primary care. By novice researchers, we mean Master’s students and junior researchers in primary care as well as experienced quantitative researchers who are engaging in qualitative research for the first time. As primary care is an interprofessional field, we bear in mind that our readers have different backgrounds, e.g. general practice, nursing, maternity care, occupational therapy, physical therapy and health sciences. This series is not a straightforward ‘cookbook’ but a source to consult when engaging in qualitative research. We neither explain all the details nor deliver an emergency kit to solve the sort of problems that all qualitative researchers encounter at least once in their lifetimes, such as failing audio recorders. We do focus on topics that have evoked a lot of questions and worries among novice researchers; the so-called frequently asked questions (FAQs).

We aim to provide researchers with practical guidance for doing qualitative research. For the journal’s editorial policy, it will serve as a standard for qualitative research papers. For those who are not involved in qualitative research on a daily basis, this series might be used as an introduction to understanding what high-quality qualitative research entails. This way, the series will also provide readers, reviewers and editors with references to criteria and tools for judging the quality of papers reporting on qualitative research.

## Further education and reading

As in quantitative research, qualitative research requires excellent methodology. Therefore, researchers in primary care need to be sufficiently trained in this type of research [2]. We hope that this series will function as a stepping stone towards participation in relevant national and international qualitative research courses or networks and will stimulate reading books and articles on qualitative research. During our supervisory work, researchers have mentioned examples of books on qualitative research that helped them in striving to perform outstanding qualitative research in primary care. Box 2 presents a selection of these books and the BMJ 2008 series on qualitative research for further reading.

Box 2. Examples of publications on qualitative research.

**Table:** 

Brinkmann S, Kvale S. Interviews. Learning the craft of qualitative research interviewing. 3rd ed. Sage: London; 2014.
^a^Bourgeault I, Dingwall R, de Vries R. The SAGE handbook of qualitative methods in health Research. 1st ed. Sage: London; 2010.
Creswell JW. Qualitative research design. Choosing among five approaches. 3rd ed. Sage: Los Angeles (CA); 2013.
^a^Denzin NK, Lincoln YS. The SAGE handbook of qualitative research. 4th ed. Sage: London; 2011.
Gray DE. Doing research in the real world. 3rd ed. Sage: London; 2013.
Holloway I & Wheeler S. Qualitative research in nursing and healthcare. 3rd ed. Wiley-Blackwell: Chichester; 2010.
^a^Miles MB, Huberman AM, Saldana J. Qualitative data analysis. A methods sourcebook. 3rd ed. Sage: Los Angeles (CA); 2014.
Morgan DL, Krueger RA. Focus group kit. Volumes 1–6. Sage: London; 1997.
Polit DF & Beck CT. Nursing Research: Generating and assessing evidence for nursing practice. 10th ed. Lippincott, Williams & Wilkins: Philadelphia (PA); 2017.
Pope C, Van Royen P, Baker R. Qualitative methods in research on healthcare quality. Qual Saf Health Care 2002, 11: 148–152.
Salmons J. Qualitative online interviews. 2nd ed. Sage: London; 2015.
Silverman D. Doing qualitative research. 4th ed. Sage: London; 2013.
Starks H, Trinidad SB. Choose your method: A comparison of phenomenology, discourse analysis and grounded theory. Qual Health Res 2007;17:1372–1380.
Tracy SJ. Qualitative quality: Eight ‘big-tent’ criteria for excellent qualitative research. Qual Inq 2010; 16(10):837–851.
BMJ series on qualitative research, published online 7 August 2008: Kuper A, Reeves S, Levinson W. An introduction to reading and appraising qualitative research. BMJ 2008;337:a288. Reeves S, Albert M, Kuper A, Hodges BD. Why use theories in qualitative research? BMJ 2008; 337:a949. Hodges BD, Kuper A, Reeves S. Discourse analysis. BMJ 2008;337:a879. Kuper A, Lingard L, Levinson W. Critically appraising qualitative research. BMJ 2008;337:a1035. Reeves S, Kuper A, Hodges BD. Qualitative research methodologies: ethnography. BMJ 2008;337:a1020. Lingard L, Albert M, Levinson W. Grounded theory, mixed methods, and action research. BMJ 2008;337:a567.

^a^ For advanced learning.

## Outline of the series

This series consists of four articles to be published consecutively in the *European Journal of General Practice*. The second article addresses FAQs about context, research questions, and designs. The third article deals with FAQs about sampling, data collection and analysis, and the last article focuses on trustworthiness and publishing qualitative research.
